# Cytogenomic epileptology

**DOI:** 10.1186/s13039-022-00634-w

**Published:** 2023-01-05

**Authors:** Ivan Y. Iourov, Alexandr P. Gerasimov, Maria A. Zelenova, Natalya E. Ivanova, Oksana S. Kurinnaia, Yulia M. Zabrodskaya, Irina A. Demidova, Evgeny R. Barantsevich, Kirill S. Vasin, Alexey D. Kolotii, Vseslav V. Ushanov, Darya A. Sitovskaya, Timur B.-A. Lobzhanidze, Maria E. Iuditskaia, Nikita S. Iakushev, Muslim M. Zhumatov, Svetlana G. Vorsanova, Konstantin A. Samochernyh

**Affiliations:** 1 Yurov’s Laboratory of Molecular Genetics and Cytogenomics of the Brain, Mental Health Research Center, Moscow, Russia; 2 Vorsanova’s Laboratory of Molecular Cytogenetics of Neuropsychiatric Diseases, Veltischev Research and Clinical Institute for Pediatrics and Pediatric Surgery of the Pirogov Russian National Research Medical University of the Russian Ministry of Health, Moscow, Russia; 3 Department of Medical Biological Disciplines, Belgorod State University, Belgorod, Russia; 4 Research Laboratory of Pediatric Neurosurgery, Polenov Neurosurgical Institute, Almazov National Medical Research Centre, Saint Petersburg, Russia; 5 Scientific Department of Polenov Neurosurgical Institute, Almazov National Medical Research Centre, Saint Petersburg, Russia; 6 Research Laboratory of Pathomorphology of the Nervous System, Almazov National Medical Research Centre, Saint Petersburg, Russia; 7 Postgraduate Neurology and Manual Medicine Department, Pavlov First Saint-Petersburg State Medical University, Saint Petersburg, Russia; 8 Department of Neurosurgery, Almazov National Medical Research Centre, Saint Petersburg, Russia; 9 Saint Petersburg State Pediatric Medical University, Saint Petersburg, Russia; 10 Polenov Neurosurgical Institute, Almazov National Medical Research Centre, Saint Petersburg, Russia

**Keywords:** Brain, Epilepsy, Chromosomal abnormalities, Chromosome instability, Copy number variants, Cytogenomics, Epileptology, Molecular cytogenetics, Molecular neurocytogenetics, Pathways

## Abstract

Molecular cytogenetic and cytogenomic studies have made a contribution to genetics of epilepsy. However, current genomic research of this devastative condition is generally focused on the molecular genetic aspects (i.e. gene hunting, detecting mutations in known epilepsy-associated genes, searching monogenic causes of epilepsy). Nonetheless, chromosomal abnormalities and copy number variants (CNVs) represent an important part of genetic defects causing epilepsy. Moreover, somatic chromosomal mosaicism and genome/chromosome instability seem to be a possible mechanism for a wide spectrum of epileptic conditions. This idea becomes even more attracting taking into account the potential of molecular neurocytogenetic (neurocytogenomic) studies of the epileptic brain. Unfortunately, analyses of chromosome numbers and structure in the affected brain or epileptogenic brain foci are rarely performed. Therefore, one may conclude that cytogenomic area of genomic epileptology is poorly researched. Accordingly, molecular cytogenetic and cytogenomic studies of the clinical cohorts and molecular neurocytogenetic analyses of the epileptic brain appear to be required. Here, we have performed a theoretical analysis to define the targets of the aforementioned studies and to highlight future directions for molecular cytogenetic and cytogenomic research of epileptic disorders in the widest sense. To succeed, we have formed a consortium, which is planned to perform at least a part of suggested research. Taking into account the nature of the communication, “cytogenomic epileptology” has been introduced to cover the research efforts in this field of medical genomics and epileptology. Additionally, initial results of studying cytogenomic variations in the Russian neurodevelopmental cohort are reviewed with special attention to epilepsy. In total, we have concluded that (i) epilepsy-associated cytogenomic variations require more profound research; (ii) ontological analyses of epilepsy genes affected by chromosomal rearrangements and/or CNVs with unraveling pathways implicating epilepsy-associated genes are beneficial for epileptology; (iii) molecular neurocytogenetic (neurocytogenomic) analysis of postoperative samples are warranted in patients suffering from epileptic disorders.

## Introduction

The last decade has seen a large number of achievements in genetics or genomics of epilepsy. Probably, genomic studies of epileptic disorders have demonstrated one of the most successful explorations of monogenic causes in a heterogeneous group of diseases. These data have been extensively used for understanding molecular mechanisms and developing treatments for this devastative condition [1, 2]. However, in contrast to monogenic epilepsies, epileptic disorders caused by chromosomal aberrations are rarely addressed. Simple querying in browseable scientific databases (e.g. https://pubmed.ncbi.nlm.nih.gov/ or https://scholar.google.com/) demonstrates a bias towards monogenic epilepsies.

Cytogenomic variations (i.e. chromosomal abnormalities and copy number variants or CNVs) are generally addressed by advanced molecular cytogenetic techniques for scanning chromosomal/subchromosomal/intragenic imbalances (array comparative genomic hybridization (CGH) or SNP array) during analysis of neurodevelopmental cohorts (i.e. cohorts of children with intellectual disability, autism, epilepsy and/or congenital malformations) [3–8]. These studies generally focus on disentangling the genomic sources for epilepsy as a symptom [3, 9]. Additionally, searching for CNVs associated with idiopathic neurodevelopmental disorders allows the determination of causative variations in epileptic cases [10–12]. Therefore, it is not surprising that cytogenomic variations manifesting as individual CNVs or CNV burdens are more profoundly studied as to chromosomal abnormalities in the molecular genetic context.

It has long been demonstrated that numerous chromosomal disorders/syndromes exhibit epileptic seizures [13]. However, molecular definition of loci and intracellular pathways affected by chromosomal aberrations remain usually elusive in the epileptic context. It is reasonable to suggest that genomic complexity of chromosomal rearrangements, which encompass from tens to hundreds of genes, hinders the possibility of uncovering molecular and cellular pathways to epilepsy in each affected individual. Since this sophistication leads to difficulties in developing the treatment of epilepsy, clinical interest is limited in cases of epileptic chromosomal abnormalities. Consequently, a large number of patients with chromosomal disorders and epilepsy cannot get appropriate care and treatment. To solve the problem, specific interpretational/bioinformatic methods are required for unraveling molecular mechanisms of epilepsy in chromosomal disorders.

Chromosomal imbalances affecting brain functioning are common and are able to involve random genomic loci of any size or even entire chromosomes (e.g. aneuploidy or gains/losses of whole chromosomes in cellular nuclei) [10, 14]. Accordingly, to describe molecular mechanisms for specific epileptic condition in an affected individual, localization and ontologies of epilepsy-associated genes as well as candidate processes for epileptiform activity are to be known.

Somatic mosaicism is another source for alterations to functioning of the central nervous system. Molecular genetic analyses have repeatedly demonstrated that tissue-specific (brain-specific) mosaicism for causative mutations is detectable in individuals with neurodevelopmental diseases including a wide spectrum of epileptic disorders [15–18]. Generally, epilepsy is associated with the presence of cellular population affected by a mutation (gene mutation) and cellular population with the same mutation in the affected brain. More precisely, abnormal cells are more likely to be concentrated in epilepsy-associated brain lesions [19, 20]. On the other hand, as shown by a series of studies of the diseased brain (neurocytogenetic or neurocytogenomic studies), a broad spectrum of brain diseases (psychiatric, neurodegenerative and neurobehavioral diseases) is shown to be associated with aneuploidy, structural chromosome abnormalities, CNVs, and genome/chromosome instability (for review, see [21–26]). Furthermore, the levels of mosaicism and rates of chromosome/genome instability generally increase through ontogeny [27–29]. These aspects of dynamic behavior of cellular genomes have not been addressed in epilepsy. In total, it seems that there is need for selecting numerous targets for cytogenomic analyses of the brain in individuals suffering from epilepsy.

A brief look at cytogenomics of epilepsy or, as we prefer to call it, cytogenomic epileptology allows an intermediate conclusion that there are several key questions, which are required to be answered to get new insights into chromosomal mechanisms and molecular/cellular pathways of epileptic disorders. We intend this communication to serve a first step forward to the answers. Since a number of previous consortium efforts in genomic research of epilepsy were recognized as successful [30], we decided to form a consortium dedicated to cytogenomic epileptology gathering a number of experts in cytogenomics and genetics of epilepsy. Our theoretical work and review of previously reported (preliminary) data are presented here-below.

### Cytogenomic variations: chromosomal abnormalities and beyond

Swimming in an ocean of articles describing genetic defects in epilepsy, one may distinguish a proportion of reports describing cases of chromosomal aberrations in individuals with epileptiform activity. However, the overwhelming majority of these cases are applicable for epilepsy research in clinical context only. Taking into account the importance of technological aspects for cytogenetic case reports (i.e. banding resolution (articles before 1990s), specificity of molecular cytogenetic methods etc. [31]), it was decided to skip detailed exploration of case reports on chromosome abnormalities in epilepsy. Recurrence of associations between chromosomal imbalance or microdeletion/microduplication syndrome and epilepsy, confirmation of the association, and application of cytogenomic techniques (e.g. array CGH or more advanced techniques) were used as criteria for detailed analysis. Table 1 summarizes data on chromosomal and subchromosomal imbalances [32–68], which correspond to these criteria.

**Table 1:** Cytogenomics of epilepsy: chromosomal imbalances

Chromosomal locus/loci	Syndrome/Aberration	References
1p36	1p36 deletion syndrome	[32, 33]
1q41q42	1q41-q42 deletion syndrome	[34]
2p16.1p15	2p16.1-p15 microduplication syndrome	[35]
3q29	3q29 duplication syndrome	[35, 37]
4p	Wolf-Hirschhorn syndrome	[38]
5q14.3	5q14.3 Deletion Syndrome	[38]
6	6q microdeletions	[40]
7q11.23	Williams-Beuren region duplication syndrome	[41]
8q21.13-q22.2	8q21.13-q22.2 duplication	[42]
8q24.3	8q24.3 duplication	[43]
9q33q34	9q33-q34 microdeletion	[44–46]
	9q33-q34 microduplication	
9q34.11	9q34.11 deletions	[47]
12q22.q23.3	De novo duplication	[48]
14q12	Duplications encompassing *FOXG1*	[49]
14qter	Ring chromosome 14	[50, 51]
15q11.1-15q13.3	Prader-Willi syndrome	[52]
	Angelman syndrome	[53]
15q13.3	15q13.3 microdeletion syndrome	[54, 55]
15q14	15q14 deletion	[56]
15q24	15q24.1 microdeletion and 15q24.2q24.3 duplication	[57]
16p13.11	16p13.11 deletion	[58]
17p13.3	Miller-Dieker Syndrome	[59]
17q12	17q12 duplication	[60]
18p	18p deletions	[61]
19p13.13	19p13.13 deletions	[62]
20	Ring chromosome 20	[63]
22q11.2	22q11.2 deletion	[64]
22q13.3	22q13.3 deletion	[65]
Xq13.1	Xq13 duplication	[66]
Xp22.13	Mosaic CDKL5 deletion (+ inversion)	[67]
Xq28	Microdeletion forms of Rett syndrome	[68]

Certainly, the table does not demonstrate the whole spectrum of recurrent cytogenomic findings in epilepsy. Still, it gives an overview of the amount of chromosomal syndromes associated with structural chromosomal imbalances and epilepsy. Additionally, individuals with aneuploidy syndromes may exhibit epileptiform activity from case to case [13, 14]. In this light, one should keep in mind somatic chromosomal mosaicism, which is able to change significantly clinical manifestation of chromosomal syndromes or to result into non-syndromic phenotypes, which, nevertheless, include epilepsy as a symptom [21, 69–71]. This suggestion becomes even more intriguing when tissue-specific or brain-specific mosaicism is proposed as a mechanism for brain dysfunction [14, 70, 71]. Thus, somatic chromosomal mosaicism with a special attention to brain-specific mosaics (structural rearrangements and aneuploidy confined to the brain) should be considered as a target for forthcoming studies in cytogenomic epileptology.

CNVs are a common type of cytogenomic variations repeatedly explored in epilepsy. The data on CNVs in epilepsy is found valuable for gene hunting and assessment of mutational (CNV) burden, which is able to cause the devastative condition. Usually, large consortia are focused on these cytogenomic variations to compare specific CNVs or CNV burdens between different patient groups [10, 72]. As a result, it becomes possible to generate big data on genomic variability and its association with variable phenotypes (i.e. cross-disorder dosage sensitivity of genomic variations) [73]. Unfortunately, replicability of these studies is poor suggesting further enlargement of acquired data sets only. Alternatively, keeping in mind a paradigm of personalized medicine, which is also applicable to epilepsy [74], one may propose individual approaches to analyze CNVs in individuals suffering from epileptic disorders. In fact, a bioinformatic concept of CNVariome might help in narrowing the outcomes of CNVs in epilepsy. This concept is based on an idea that the whole set of CNVs in an individual shape the phenotype. Accordingly, all CNVs detected in a patient are viewed as a system, where CNVs are elements interacting with each other through ontologies of genes affected by these cytogenomic variations [75]. Using this concept, one may uncover molecular and cellular processes changed by CNVs in an individual. The application of CNVariome concept for studying epilepsy has the potential to highlight new mechanisms of this devastative condition.

As one may see from the Table 1, imprinting disorders are associated with chromosome imbalances (deletions at 15q11.1-15q13.3) and epilepsy. Indeed, the two best known imprinting disorders—Angelman and Prader-Willi syndromes—represent a major focus of genetic epileptologists [76]. Here, it is noteworthy that runs of homozygosity or long contiguous stretches of homozygosity spanning shortly the imprinted loci (detectable by SNP array) are associated with epilepsy in atypical cases of Angelman or Prader-Willi syndrome [77, 78]. However, additional research is needed for defining phenotypic outcomes of these cytoepigenomic variations.

Another type of cytogenomic variations poorly addressed in epilepsy is referred to chromosome (genome) instability. An appreciable number of neurological and psychiatric diseases are associated with chromosome instability [24]. Moreover, chromosomal imbalances (deletions, duplications, ring chromosomes) and CNVs are able to produce chromosomal instability in cases demonstrating epileptiform activity [79, 80]. For instance, a specific type of chromosomal inability (chromohelkosis or chromosome ulceration/wound) is relatively common in neurodevelopmental cohorts, which include individuals with epilepsy (for more details, see [80]). In addition, it is pertinent to mention that brain-specific chromosome and genome instability is a key element of the pathogenetic cascades for several brain diseases [21, 24]. Consequently, it appears important to test postoperative and postmortem samples from individuals with epileptic disorders in the chromosome instability context.

**Fig. 1:**
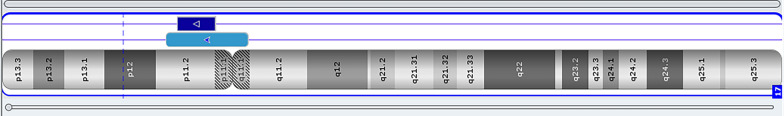
SNP array analysis of a derivative chromosome 17 demonstrating the co-occurrence of mosaic and non-mosaic chromosomal abnormality (chromohelkosis)

**Fig. 2:**
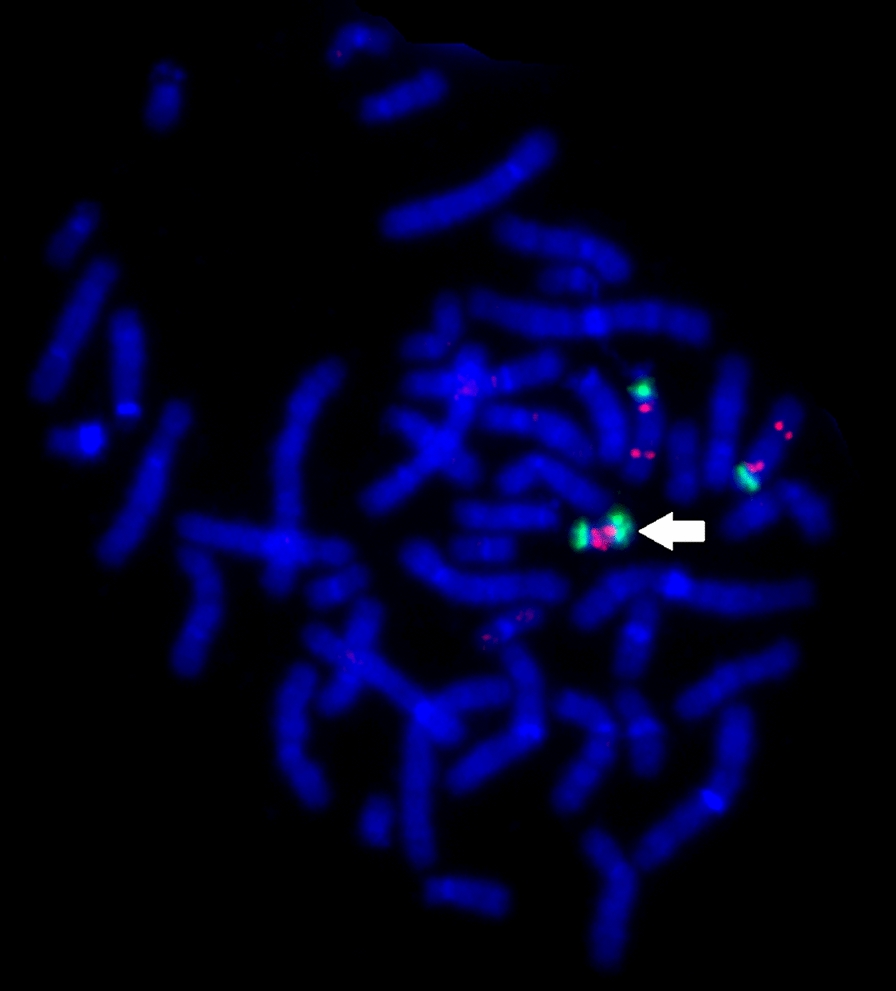
Two-color-FISH demonstrating the presence of supernumerary rearranged (inv dup shaped) chromosome 15 (white arrow) in a child with epilepsy (DNA probes: SpectrumOrange—SNRPN + PML; SpectrumGreen—CEP15 or D15Z1)

Finally, cytogenomic views on epilepsy are incomplete without considering small supernumerary marker (rearranged) chromosomes. Clinical outcomes of these chromosomal imbalances are highly heterogeneous ranging from normal to severe phenotypes (including epilepsy). Structural variability is supposed to be essential mechanism for such a phenotypic heterogeneity [81]. Another source for the heterogeneity is mosaicism [82]. Figure 1 demonstrates SNP array analysis of a mosaic case of supernumerary rearranged chromosome 17 in a child with epilepsy (Fig. 1). Alternatively, common types of small supernumerary marker chromosomes may even cause clinically recognizable syndromes exhibiting epilepsy. Probably, one of the best example of such syndromes is the inv dup(15) syndrome [83]. Figure 2 depicts fluorescence in situ hybridization (FISH) analysis of this syndrome in a child suffering from a severe form of epilepsy (Fig. 2). In total, structural and phenotypic heterogeneity of small supernumerary marker chromosomes requires systematic analysis for the clinical interpretation. Databases may help epileptologists and clinical geneticists to assess contribution of small supernumerary marker chromosomes to the etiology of epilepsy. The most detailed information concerning associations between epilepsy and supernumerary marker chromosomes may be acquired using the database of marker chromosomes managed by Prof. Thomas Liehr (http://cs-tl.de/DB/CA/sSMC/0-Start.html). In summary, supernumerary marker chromosomes should be kept in mind when cytogenomic epileptology studies are performed.

To use cytogenomic data for unraveling mechanisms of epileptiform activity, specific bioinformatic methods are required. More precisely, chromosomal abnormalities and CNVariome (individual set of CNVs) are to be processed by techniques allowing the analysis of large gene sets. Fortunately, there are specific methods for ontology- and pathway-based evaluation of genes affected by chromosomal imbalances/CNVs based on data fusion and systems analysis [84–87]. These methods are effective enough to provide therapeutic opportunities in patients with chromosomal abnormalities, which are considered as genetic defects associated with untreatable conditions [88]. Since genes are essential elements in systems developed by processing cytogenomic data, it seems logical to address epilepsy associated genes in the pathway context.

### Epilepsy genes, pathway-based analysis (classification) and candidate processes

Using a variety of gene hunting strategies, numerous epilepsy-associated genes have been identified during the last decades. Then, molecular processes or pathways implicating these genes have been described [1, 2, 10, 72, 73, 89]. Table 2 shows pathways implicating epilepsy-associated genes or gene families and corresponding disorders.

**Table 2:** Essential types of pathways implicating epilepsy-associated genes (gene families) and corresponding epileptic disorders

Type of pathways	Epileptic disorders	Chromosomal loci	Genes
Sodium channels	Developmental and epileptic encephalopathy, 6 (Dravet and non-Dravet types), 11, 13, 52, 62 types; generalized epilepsy with febrile seizures plus, types 1, 2, 7; familial focal epilepsy with variable foci 4; familial febrile seizures 3A and 3B; benign familial infantile seizures 3, 5 types	2q24.3	*SCN1A, SCN2A, SCN3A, SCN7A/SCN6A, SCN9A*
		3p22.2	
		11q23.3	
Potassium channels	Developmental and epileptic encephalopathy, 7, 14, 24, 57 types; generalized epilepsy with febrile seizures plus, type 10; epilepsy, progressive myoclonic 3, with or without intracellular inclusions; myokymia; benign neonatal seizures, type 1 and 2; cerebellar atrophy, developmental delay, and seizures; Liang-Wang syndrome; paroxysmal nonkinesigenic dyskinesia, 3, with or without generalized epilepsy; epilepsy, intellectual/developmental delay	1p13.2-1p13.3	*KCN2A, KCNT2, HCN1, KCNT2, KCNB1, KCNQ1, KCND3, KCNC4, KCNA10*
Calcium channels	Developmental and epileptic encephalopathy, 42 and 69 types; primary aldosteronism, seizures, and neurologic abnormalities; susceptibility to childhood absence epilepsy 6; susceptibility to idiopathic generalized epilepsy 6 and 9; susceptibility to juvenile myoclonic epilepsy 6	3p21.31-3p14.3	*CACNA1E, PACS2, CACNA1A, CACNA1D, CACNA2D2, CACNA2D3, CACNG6, CACNG7, CACNG8*
		19q13.42	
Chloride channels	Susceptibility to idiopathic generalized epilepsy, 11; susceptibility to juvenile absence epilepsy, 2; susceptibility to juvenile myoclonic epilepsy, 8	Wide cytogenetic distribution	*CLCN* family
Na/K^+^ pump	Hypomagnesemia, seizures, and mental retardation 2, Developmental and epileptic encephalopathy 98, 99 and 104	1q23.2-1q24.2	*ATP6V1A, PIGB, ATP1A2, ATP1A3, ATP1A2, ATP1A4, ATP1B1, KCNJ10, LY9, FXYD2, FXYD6, HEPACAM, FXYD1, FXYD5, FXYD7*
		11q23.3- 11q24.2	
		19q13.12-19q13.2	
GABA receptors	Developmental and epileptic encephalopathy 19, 43, 45, 54, 74, 78, 79 types; infantile or early childhood epileptic encephalopathy, 2; familial febrile seizures 8; generalized epilepsy with febrile seizures plus, type 3; susceptibility to generalized epilepsy with febrile seizures plus, type 5; susceptibility to childhood absence epilepsy 4, 5; susceptibility to juvenile myoclonic epilepsy, 5	4p12	*GABRA2, GABRB1, GABRB2, GABRA1, GABRG2, GABBR2, GABRB3, GABRA5*
		5q34	
Glycine receptors	Glycine encephalopathy with normal serum glycine	Genes of glycine system have wide cytogenetic distribution	
NMDA receptors	Developmental and epileptic encephalopathy 27 and 46 types; neurodevelopmental disorder with or without hyperkinetic movements and seizures, autosomal dominant and autosomal recessive types; focal epilepsy with speech disorder and with or without impaired intellectual development; intellectual developmental disorder, autosomal dominant 6, with or without seizures	9q31.1-9q34.3	*GRIN1, GRIN2B*, *GRIN2D, GRIN1, GRIN3A, NSMF*

From the cytogenomic point of view, one may notice cytogenetic co-localization of epilepsy-associated genes from same gene families. This observation is important for deciphering the role of novel chromosomal rearrangements and large CNVs (> 100–150 kb) encompassing these loci in the epilepsy etiology. Epilepsy-associated gene clustering allows us to suggest that intranuclear interactions between these chromosomal loci through specific nuclear genome organization exist. In its turn, such kind of nuclear organization of epilepsy-associated genes may be involved in regulation/deregulation of the clusters (discussed hereafter).

Alternatively, looking at epilepsy-associated gene loci in the disease context (e.g. specific autosomal dominant epilepsy subtypes), the contrary is observed: variable localization and implication in molecular pathways of genes associated with the same type of autosomal dominant epilepsy (Table 3). Thus, we have to recognize the extended complexity of cytogenomic and “pathwayomic” parameters of epilepsy-associated genes.

**Table 3:** Chromosomal loci and genes associated with autosomal dominant lateral temporal lobe epilepsy and autosomal dominant nocturnal frontal lobe epilepsy

Chromosomal loci	Phenotype	Disease MIM*	Gene/Locus	Gene/Locus MIM	Gene function
*Autosomal dominant lateral temporal lobe epilepsy*					
3q25-q26	Epilepsy, familial temporal lobe, 6	615697	*ETL6*	–	–
4q13.2-q21.3	Epilepsy, familial temporal lobe, 3	611630	*ETL3*	–	–
7q22.1	Epilepsy, familial temporal lobe, 7	616436	*RELN*	600514	Neuronal migration
8q13.2	Epilepsy, familial temporal lobe, 5^	614417	*CPA6*	609562	Carboxypeptidase
9q21-q22	Epilepsy, familial temporal lobe, 4	611631	*ETL4*	–	–
10q23.33	Epilepsy, familial temporal lobe, 1	600512	*LGI1*	604619	Glutamate system
11q13.2	Epilepsy, familial temporal lobe, 8	616461	*GAL*	137035	Neuropeptide
12q22-q23.3	Epilepsy, familial temporal lobe, 2	608096	*ETL2*	–	–
*Autosomal dominant nocturnal frontal lobe epilepsy*					
1q21.3	Epilepsy, nocturnal frontal lobe, 3**	605375	*CHRNB2*	118507	Nicotinic acetylcholine receptor beta-2 subunit
8p21.2	Epilepsy, nocturnal frontal lobe, type 4	610353	*CHRNA2*	118502	Neuronal nicotinic cholinergic receptor alpha-2 subunit
9q34.3	Epilepsy nocturnal frontal lobe, 5	615005	*KCNT1*	608167	Sodium-activated potassium channel
15q24	Epilepsy, nocturnal frontal lobe, type 2	603204	*ENFL2*	–	–
20q13.33	Epilepsy, nocturnal frontal lobe, 1	600513	*CHRNA4*	118504	Neuronal nicotinic acetylcholine receptor alpha-4 subunit

^*^—Mendelian inheritance in Man (https://omim.org/); ^—autosomal recessive inheritance is reported, as well; **—autosomal dominant inheritance is uncertain;

Ontologies of epilepsy-associated genes have been systematically used for uncovering disease-causing pathways [1, 2]. On the other hand, participation of these genes in same gene families and molecular pathways (Table 2) is used as a successful gene hunting strategy [90]. Nonetheless, current knowledge about molecular and cellular systems, which functioning is mediated by a myriad of pathways, implies to apply pathway-based classification for the definition of disease mechanisms [91]. The availability of bioinformatic tools for solving this task in cases of gene mutations [92] and chromosomal aberrations [93] simplifies classifying diseases according to molecular and cellular pathways. Thus, for uncovering the way from genomic changes to epileptic phenotype passing through pathways or metabolic processes, classification issues should be addressed. Consequently, it is unavoidable to establish correlations between (cyto)genomic and clinical (phenotypical) data or to establish genotype–phenotype correlations.

### Classification matters

The essential classification of epilepsy is based on clinical observations (as for the overwhelming majority of complex diseases). ILAE (International League Against Epilepsy) classification of the epilepsies is the basic document [94]. Clinical and diagnostic practice (including genetic testing) in epileptology is performed using the classification. Genetic classification of epilepsy, which is less official than clinical and is closer to nature, is almost completely dedicated to monogenic forms/syndromes [95]. Thus, 977 epilepsy-associated genes were classified according to the clinical outcomes of the mutations/variants. Four categories were proposed [96]: (1) gene mutations causing epilepsy per se or syndromes with epilepsy as the core symptom; (2) gene mutations causing neurodevelopmental anomalies/malformations resulting in epilepsy; (3) gene mutations causing gross systemic abnormalities accompanied by epilepsy; (4) gene variants of uncertain significance. In summary, it seems that neither cytogenomic variations nor disease pathways are the focus for classification of epilepsy. Consequently, we conclude that a large bioinformatic, clinical and molecular cytogenetic (cytogenomic) work is required to fill this gap in epileptology, (cyto)genomic epileptology.

### Preliminary cytogenomic analysis of the cohort

To form the cohort for cytogenomic analysis of epilepsy, we have initially selected individuals from the Russian neurodevelopmental cohort. Once selected, molecular karyotyping by array CGH or SNP array analyses has been performed. Details and cohort description have been previously presented elsewhere [7, 12, 78, 97–99]. Tables 4 and 5 present the data.

**Table 4:** Gross chromosomal aberrations detected in children with epilepsy forming the neurodevelopmental cohort

Chromosome abnormality according to cytogenetic analysis	Chromosomal loci according to SNP array data	Aberration (copy number change)	Brief clinical description
46,XX,add(3)(p26)	3p26.3	× 1	Developmental delay, epilepsy, unsteady gait, developmental abnormalities: broad flattened face, cleft palate, short toes, sandal gap, syndactyly of II-III toes; structural heart defect
	3p26.3p24.3	× 3	
47,XX, + mar	17p11.2q11.1	× 2 ~ 3	Developmental delay, epilepsy, biliary dysfunction, hypertelorism of the palpebral fissures, congenital clouding of the cornea of the right eye, strabismus, wide nose, low-lying auricles, ear appendages on the left; long QT, increase in mobility, volume and changed parenchyma of the kidneys
	17p11.2	× 3	
46,XX,der(11)?add(11)(p13)ins(11)(p13q21q23.3)	–	–	Developmental delay, epilepsy, developmental abnormalities: up-slanting palpebral fissures epicanthus, broad nasal bridge, epithelial coccygeal passage; congenital heart and celiac diseases
46,XX,del(6)(q22.?2q23.?3)	6q22.1q23.2	× 1	Developmental delay, epilepsy, developmental abnormalities: thin sparse hair, narrow face, hypotelorism of the palpebral fissures, enlarged middle part of the face, retrognathia, dys-plastic auricles, small teeth, brachydactyly, thin nails, thoracic kyphosis
46,XY,del(15)(q11.2q1?3)	15q11.2q13.1	× 1	Developmental delay, epilepsy, developmental abnormalities: flattened face, high forehead, ocular hypotelorism, high-arched palate, short neck, wobbly gait

**Table 5:** CNVs detected in children with epilepsy forming the neurodevelopmental cohort

Genetic sex	Copy number	Chromosome locus (loci)
*Chromosome X*		
XX	× 3	Xp22.13
	× 3	Xq27.3
	× 3	Xq28
	× 1	Xq23
	× 2 ~ 3	Xq26.2q26.3
	× 3	Xq22.1
	× 0	Xp11.23
XY	× 2	Xq28
	× 2	Xp22.13
	× 2	Xq21.1
	× 0	Xq21.1
	× 2	Xp22.31
	× 2	Xp11.4
	× 2	Xq27.3
	× 2	Xp11.23
	× 2	Xq12
*Chromosome Y*		
XY	× 2	Yq11.223
	× 2	Yq11.223q11.23
	× 0	Yq11.23
*Chromosome 1*		
XY	× 1	1q42.13
	× 1	1p31.1
	× 1	1p22.1
	× 1	1p13.2
XX	× 3	1p36.32
	× 4	1p31.3
	× 3	1p21.3
	× 1	1p21.1
*Chromosome 2*		
XX	× 4	2q22.1
XY	× 1	2q37.1
	× 1	2q24.3q31.1
		2q24.3
	× 1	2q31.1
	× 3	2p12
*Chromosome 3*		
XY	× 3	3p25.3
	× 1	3p14.1
	× 4	3q29
XX	× 3	3p26.3
XX	× 1	3p26.2
	× 1	3p14.2
	× 1	3q23
	× 4	3q26.33
*Chromosome 4*		
XX	× 3	4q34.3
XY	× 4	4q21.21
	× 3	4q31.3
	× 1	4q21.22
*Chromosome 5*		
XX	× 3	5q13.3
	× 1	5q22.2
	× 3	5p13.2
	× 1	5q13.2
	× 1	5q33.1
*Chromosome 6*		
XX	× 1	6p11.2
	× 1	6q25.3
XY	× 3	6q26
*Chromosome 7*		
XY	× 1	7p12.3
	× 3	7p21.1
	× 3	7p13
	× 1	7q21.2
XX	× 3	7p22.3p21.2
	× 1	7q32.3
	× 1	7q11.21
	× 4	7q21.11
	× 1	7q31.1
	× 1	7q22.1
*Chromosome 8*		
XY	× 3	8p23.3
	× 1	8q12.2
	× 1	8p21.3
	× 1	8p21.2
	× 4	8q21.13
	× 1	8p23.1
	× 1	8q12.1
*Chromosome 9*		
XY	× 1	9q34.3
XX	× 1	9q34.3
	× 4	9q21.31
	× 4	9q33.2
	× 1	9q34.13
	× 3	9q34.12
	× 2 ~ 3	9p24.3p24.2
	× 3	9p24.3
	× 1	9p24.3p23
	× 1	9p23
*Chromosome 10*		
XY	× 3	10q24.32
	× 4	10q24.32
	× 3	10q24.1
	× 4	10q25.2
	× 4	10p12.31
	× 4	10q26.3
	× 1	10q25.1
	× 4	10p15.3
	× 3	10q24.2
*Chromosome 11*		
XY	× 1	11p15.5
	× 1	11p13
	× 4	11p12
XX	× 1	11p15.4
	× 1	11q22.3
	× 3	11q22.3
	× 4	11p13
	× 3	11q13.1
	× 3	11q12.1
*Chromosome 12*		
XX	× 1	12q24.13
	× 3	12p13.31
	× 1	12p12.1
	× 1	12q13.12
XY	× 1	12q13.13
	× 3	12p13.31
	× 1	12q24.31
	× 1	12p12.2
*Chromosome 13*		
XY	× 1	13q12.12
XX	× 1	13q33.3
	× 3	13q14.11
	× 1	13q33.3q34
*Chromosome 14*		
XY	× 1	14q24.1
	× 1	14q21.3
*Chromosome 15*		
XX	× 1	15q21.3
	× 1	15q26.3
	× 1	15q15.3
	× 1	15q21.1
XY	× 1	15q15.1
	× 1	15q21.3
	× 1	15q22.2
	× 3	15q26.3
	× 1	15q11.2
*Chromosome 16*		
XY	× 1	16p13.3
	× 1	16q23.2
XX	× 3	16p13.3
	× 1	16p13.3
	× 1	16p11.2
	× 3	16q24.3
	× 1	16p13.12
	× 1	16q23.1
	× 1	16q23.3
	× 1	16q24.3
*Chromosome 17*		
XY	× 3	17p13.3
	× 1	17q21.1
	× 2 ~ 3	17p13.2p12
	× 4	17p13.2p13.1
	× 3	17p13.1
	× 3	17p11.2
	× 1	17p13.2
XX	× 3	17p13.3
	× 1	17q21.31
	× 1	17q21.1
*Chromosome 18*		
XX	× 3	18q12.1
	× 3	18q21.2
*Chromosome 19*		
XY	× 1	19p13.3
	× 3	19p13.11
XX	× 1	19q13.2
	× 1	19q13.33
	× 3	19q13.41
	× 3	19p13.12
*Chromosome 22*		
XY	× 4	22q13.2
	× 2 ~ 3	22q11.1q11.22
	× 3	22q11.21
	× 3	22q13.33
	× 2 ~ 3	22q11.1q11.23
XY	× 3	22q11.21

Gross chromosome rearrangements were detected by cytogenetic analysis in 5 (~ 2%) out of 300 individuals. Four cases were confirmed by molecular karyotyping. Certainly, further analysis of actual and extended cohort would show additional cases of chromosomal abnormalities associated with epilepsy, which would demonstrate new pathways implicated in the pathogenesis after bioinformatic analysis. Additionally, molecular karyotyping has allowed us the section of 153 CNVs, which might be implicated in epilepsy pathogenesis in our cohort (Table 5). Currently, in silico analysis using an original and established bioinformatic technology [75, 84–87], of these CNVs is performed.

Although the results of our cytogenetic and cytogenomic analysis of the consortium (epilepsy) cohort are extremely preliminary, we decided to share these data with the scientific community inasmuch as it helps to choose future directions in cytogenomic epileptology. It is to note that non-random sex distribution of chromosome-specific CNVs encompassing autosomal genes is observed. One may hypothesize epilepsy-specific gonosome-autosome interactions by non-random genomic loci, which potentially occur through the specificity of intranuclear chromsome/genome organization. Current bioinformatic analyses shows that a significant proportion of CNVs encompasses genes involved in following pathways: cell cycle regulation, programmed cell death, DNA reparation and replication. Among others, mTOR, PI3K-Akt, p53, PTEN, MAPK pathways have been affected. Since these pathways are associated with brain disorders including epilepsy and genome stability maintenance [24, 100–104], somatic mosaicism and chromosome (genome) instability should become an important focus of cytogenomic epileptology.

### Somatic mosaicism and chromosome instability: neurocytogenetic (neurocytogenomic) aspects

As noted before, somatic mosaicism for gene mutations is common in epilepsy and seems to play a specific role in the pathogenesis of epileptic disorders, especially when affecting brain tissues/brain foci. Genomic analyses of postoperative samples of the brain in patients suffering from epilepsy have become a common research practice [19, 20, 105, 106]. Currently, several monogenic neurodevelopmental disorders exhibiting epilepsy have been reported to demonstrate brain-specific mosaicism for gene mutations: focal cortical dysplasia—*MTOR* (1p36.22), *TSC1* (9q34.13), *TSC2* (16p13.3), *DEPDC5* (22q12.2q12.3) [107–110]; hemimegalencephaly—*MTOR* (1p36.22), *AKT3* (1q43q44), *PIK3CA* (3q26.32), *RPS6* (9p22.1), *AKT1* (14q32.33) [107, 108, 111, 112]; hypothalamic hamartoma—*GLI3* (7p14.1),*OFD1* (Xp22.2) [113, 114]; nonlesional focal epilepsy—*SLC35A2* (Xp11.23) [115]; Sturge-Weber syndrome (leptomeningeal angiomatosis)—*GNAQ* (9q21.2) [116]; tuberous sclerosis 16p13.3—(*TSC2*) [117]. As one may observe, mosaic mutations in these forms of mosaicism affect mTOR and PI3K-Akt pathways as well as pathways of cell cycle regulation and programmed cell death. Since deregulation of these pathways leads to chromosome instability in brain diseases (for review, see [24]), somatic chromosomal mosaicism and instable genome behavior at the chromosomal level are likely to be associated with epilepsy and are able to be at least elements of the epileptic pathogenic cascade.

Somatic chromosomal mosaicism and chromosome instability are common genetic defects detectable in neurodevelopmental cohorts (i.e. high rates of chromosomal mosaicism in children with idiopathic autism and intellectual disability with congenital anomalies and epilepsy) [118, 119]. Moreover, somatic mosaicism may initiate instability, which rates are variable and correlate with phenotypic dynamics (increase in rates of mosaicism/instability → worsening; decrease in rates of mosaicism/instability → improvement) [121–123]. Finally, somatic chromosomal mosaicism and chromosome/genome instability represent an important part of pathogenetic cascades of a wide spectrum of brain disorders, including neurobehavioral, neurodevelopmental, psychiatric, neurological and neurodegenerative conditions [21–25, 124–129]. Thus, cytogenomic research of chromosomal variations in the brain (neurocytogenetic or neurocytogenomic analyses) of individuals with epilepsy has the potential to bring new insights into understanding the etiology.

Genome and chromosome instability in the brain is mainly generated in early ontogeny. The developing human brain is significantly affected by chromosome instability (up to 35% of cells) [130–132]. Normally, cellular population affected by chromosome instability diminishes due to neuronal cell death [133, 134]. During later ontogenetic periods genome/chromosome instability in the brain is generally the result of genetic-environmental interactions (i.e. environmental triggers produce a genomically instable cellular population, which is initially susceptible to the instability due to mutational burden altering genome safeguarding pathways) [24, 135]. These neurocytogenetic observations allow suggesting that studies in cytogenomic epileptology require not only analysis and monitoring of chromosome instability, but also a sophisticated evaluation of genome susceptibility to the instability. If successful, neurocytogenetic (neurocytogenomic) studies are able to lead the way to developing diagnostic approaches for suggesting the presence of brain-specific epilepsy-associated genome instability (for details, see [136]) and therapeutic approaches targeted toward inhibition of brain-specific chromosome instability [137].

The most enigmatic area of neurocytogenetics or neurocytogenomics is nuclear genome organization at chromosomal level in brain diseases. It is important to note that epilepsy was the first disease, in which chromosome behavior was studied in the affected brain [138]. Unfortunately, no additional efforts in this direction were made. In fact, neurocytogenetic analysis of nuclear organization in the unaffected and diseased brain has never been systematically performed. Current molecular cytogenetics and cytogenomics possess technological possibilities to perform high-resolution analysis of chromosomal arrangements and rearrangements in post-mitotic cells of the human brain [139–142]. The analysis of brain-specific chromosomal nuclear organization appears even more attractive taking into account that spatial positioning of chromosomes determines behavior and stability of the nuclear genome in an interphase nucleus [140, 142, 143]. In the light of cytogenomic epileptology, studying chromosomal nuclear organization in postoperative brain samples of individuals with epilepsy might bring new important insights into our understanding of molecular and cellular processes leading to focal brain dysfunction.

## Conclusions

Theoretical work of our consortium has allowed us to make some important conclusions, which underlie future directions in cytogenomic epileptology:

- Cytogenomic variations require more profound research in epileptic disorders.
- More detailed bioinformatic analyses (e.g. application of CNVariome concept and systems analysis) of epilepsy-associated genes are needed in cases of chromosomal abnormalities and CNVs.
- Neurocytogenetic (neurocytogenomic) studies of chromosomal variation and instability in postoperative samples are warranted in patients suffering from epileptic disorders.
- Cytoepigenomic variations (long contiguous stretches of homozygosity spanning shortly the imprinted loci) should not be left aside in large-scale studies in epilepsy genetics.
- Supernumerary marker chromosomes are an important target for studies in cytogenomic epileptology.
- Extended complexity of cytogenomic (non-random gene co-localization and clusterization) and “pathwayomic” parameters of epilepsy-associated genes as well as behavior of chromosomal loci in interphase should be a focus of cytogenomic studies in epileptology.
- Genotype–phenotype correlations are an important part of cytogenomic studies in epileptology.
- Cytogenomic pathway-based classification of epileptic disorders seems to be useful for basic and practical research of epilepsy.
- Pathways (e.g. mTOR, PI3K-Akt, p53, PTEN, and MAPK) altered in epilepsy and associated with chromosome and genome instability require profound exploration.
- Somatic chromosomal mosaicism is a target for future studies in cytogenomic epileptology.
- Studies of chromosomal nuclear organization in postoperative brain samples of individuals with epilepsy appear to become an innovative and perspective area of biomedical research.
- The consortium focused on studying cytogenomic (cytogenetic and molecular cytogenetic) aspects of epilepsy has the potential to bring new insights in current epileptology.

Chromosomal abnormalities and CNVs represent an important, albeit poorly explored, genetic causes of epilepsy [13, 14, 21]. The problem of lacking cytogenetic and cytogenomic studies of epilepsy is likely to arise from general decrease in cytogenetic competence [144]. It has been systematically reported that ignoring chromosomal approaches to solving genomic biomedical problems lead to incomplete understanding of mechanisms for genetic diseases [144–146]. The formation of our consortium is basically aimed at incorporating cytogenomic variations to the complemented view of genetic causes of epilepsy. We have preferred to use the term “cytogenomic” for designating the consortium in its initial and established meaning [147]. Despite the fair discussions about the term of cytogenomics [148], our consortium is definitively a cytogenomic one, inasmuch as it is focused on studying genome of individuals suffering from epilepsy by molecular cytogenetic and genomic technologies in the chromosomal context. Thus, we concluded the designation “consortium on cytogenomic epilptology” to be appropriate.

## Dedication

Our communication as well as our consortium is dedicated to Professor Yuri B. Yurov, an outstanding researcher in the field of medical genomics, whose contribution to molecular cytogenetics and cytogenomics is hard to estimate [149]. His main research targets were chromosomal abnormalities in brain disorders and genomic variations in the diseased brain. Accordingly, Yuri’s original ideas and findings are consistently used for the work of the consortium.

## Data Availability

Not applicable.
